# Editorial: The use of large animal models to improve pre-clinical translational research

**DOI:** 10.3389/fvets.2022.1086912

**Published:** 2022-11-15

**Authors:** Mark Gray, Stefano Guido, Abirami Kugadas

**Affiliations:** ^1^The Royal (Dick) School of Veterinary Studies, The Roslin Institute, University of Edinburgh, Edinburgh, United Kingdom; ^2^Bioresearch and Veterinary Services, University of Edinburgh, Edinburgh, United Kingdom; ^3^Takeda Pharmaceutical Company Limited, Cambridge, MA, United States

**Keywords:** disease models (animal), comparative model, large animal models, translational, genetically altered animals

Drug and medical device research and development is an expensive and time-consuming process, involving *in vitro* target identification/validation, progressing through to phase 0, I, II, III, and IV clinical trials. It has been estimated that in the USA alone it costs between $1.8–2.6 billion and 13 years to develop and gain regulatory approval for a new drug. The situation is similar for medical devices, costing between $75–94 million and 4.5 years (1). In addition to these financial and time commitments, most drugs showing pre-clinical success fail to show efficacy in clinical trials, with an estimated failure rate of 86–95% (1, 2). These issues can deter drug companies from developing novel therapeutics.

While the use of live animals should be replaced wherever possible, animal models are still necessary to generate data in support of conducting human clinical trials. Pre-clinical research has historically focused upon *in vitro* (e.g., cell line) and *in vivo* rodent models for studying human conditions. Unfortunately, *in vitro* models are unable to faithfully recapitulate complex physiological environments. With a plethora of relatively low cost inflammatory, neoplastic, transgenic and knockout rodent models now available, they have become ingrained in pre-clinical research. Rodent models often fail to capture disease characteristics typically seen in humans, they fail to address translatable immune related outcomes, are not very useful to address specific toxicities and are unsuitable for the application of size-relevant clinical imaging (ultrasound, computed tomography, magnetic resonance imaging, bronchoscopy and endoscopy) and surgical techniques used in human patients. These issues will continue to hamper the development of new therapies and medical devices. To mitigate against these issues, over recent years there has been a scientific push for the development of translational pre-clinical large animal models that can more accurately represent the complex clinical and pathologic features of a variety of human diseases. Whether the model is predictive (treatment effects), isomorphic (similar symptoms but different etiology), or homologous (same symptoms and etiology) (3), various large animal species can be used for translational research.

Pigs, and perhaps to a lesser extent sheep and goats, have gained significant scientific attention as translational models as they share important proteomic, genomic and immunologic similarities to that of humans. Each of these species have been successfully used for cardiovascular, respiratory, gastrointestinal, immune, musculoskeletal, neurological and cancer studies. The pig has also been utilized for interspecies transplantation studies and satisfies the Food and Drug Administration (FDA) evaluation requirements for pharmaceutical drugs (4). Furthermore, similarities in anatomy and physiology allow anesthetic techniques, drug administration, advanced imaging and surgical procedures to be used in large animal species as they would be used in humans. Despite these advantages, several perceived limitations have obstructed their widespread use in biomedical studies. These issues include higher costs of maintenance and the need for specialized surgical facilities and veterinary care. Although these limitations have to be considered when developing or using large animal models, the translational data they produce can overcome the issues with rodent models and enable significant advancements in the understanding and treatment of a wide variety of disease conditions to be made.

This Frontiers in Veterinary Science Research Topic presents six Original Research Articles, three Reviews, one Case Report, one Brief Research Report, and one Methods paper which span multiple large animal species including pigs, horses, goats, and sheep.

In the original research section Qi et al. investigated methods and management strategies used in an ovine veno-arterial extracorporeal membrane oxygenation model. They suggested it could be used as a platform for pathophysiology exploration and new medical device validation. Gadomski et al. developed an ovine orthopedic model, with their findings indicating that the carpometacarpal joint may be used for investigating human foot and ankle orthopedic devices. Xu et al. investigated how to enhance the ability of immunological tolerance in pig-to-human xenotransplantations through the generation of triple-gene-modified Diannan miniature pigs. Wedlich et al. described the refinement of a goat bovine tuberculosis model using a video-guided endoscopic intra-bronchial inoculation procedure which could be used for testing tuberculosis vaccine efficacy. Höglund et al. described a protocol for isolating extracellular vesicles from the bronchio-aveolar fluid (BALF) of asthmatic horses. Sper et al. generated a novel immunodeficient pig model and demonstrated successful engraftment of swine leukocyte antigen mismatched allogeneic D42 fetal liver cells and human CD34+ hematopoietic stem cells after *in utero* cell transplantation.

In the mini review and review sections, Rose et al. describes how the pig can be used as a translational gastrointestinal microbiota model for elucidating the pathogenesis of human bowel disorders. Schaaf and Gonzalez also looked at pig gastrointestinal models, but instead focused on describing gene editing technologies to advance intestinal disease research. Billings and Anderson reviewed the role of animal models (including pig, sheep, and goats) utilized for the study of bacterial osteomyelitis, and their critically important role in understanding how the models can be used to improve bacterial osteomyelitis management.

In the case report, brief research report and methods sections, Weisskopf et al. presented work elaborating the do's and don'ts in porcine models of aortic insufficiency. Furthermore, Malbon et al. described a hemodynamically, highly relevant atrial septal defect with incompetent mitral and tricuspid valves, in an asymptomatic, juvenile pig. They suggested that although not common, congenital heart defects could influence experimental variability or mortality rates. Solanes et al. looked at porcine acute myocardial infarction models and reported the differences in infarct size and cardiac function for various anesthetic protocols and pig breeds.

## Author contributions

MG designed and formulated the idea for this special issue and recruited both co-authors. All authors listed have made a substantial, direct, and intellectual contribution to the work and approved it for publication.

## Conflict of interest

Author AK was employed by Takeda Pharmaceutical Company Limited. The remaining authors declare that the research was conducted in the absence of any commercial or financial relationships that could be construed as a potential conflict of interest.

## Publisher's note

All claims expressed in this article are solely those of the authors and do not necessarily represent those of their affiliated organizations, or those of the publisher, the editors and the reviewers. Any product that may be evaluated in this article, or claim that may be made by its manufacturer, is not guaranteed or endorsed by the publisher.
